# Deletion of the SARS-CoV-2 Spike Cytoplasmic Tail Increases Infectivity in Pseudovirus Neutralization Assays

**DOI:** 10.1128/JVI.00044-21

**Published:** 2021-05-10

**Authors:** Jingyou Yu, Zhenfeng Li, Xuan He, Makda S. Gebre, Esther A. Bondzie, Huahua Wan, Catherine Jacob-Dolan, David R. Martinez, Joseph P. Nkolola, Ralph S. Baric, Dan H. Barouch

**Affiliations:** a Center for Virology and Vaccine Research, Beth Israel Deaconess Medical Center, Harvard Medical School, Boston, Massachusetts, USA; b Ragon Institute of MGH, MIT, and Harvard, Cambridge, Massachusetts, USA; c University of North Carolina at Chapel Hill, Chapel Hill, North Carolina, USA; Emory University

**Keywords:** spike, neutralization assay, lentiviral vector, SARS-CoV-2, antibody, neutralizing antibodies

## Abstract

Pseudotyped viruses are valuable tools for studying virulent or lethal viral pathogens that need to be handled in biosafety level 3 (BSL3) or higher facilities. With the explosive spread of the coronavirus disease 2019 (COVID-19) pandemic, the establishment of a BSL2 adapted severe acute respiratory syndrome coronavirus 2 (SARS-CoV-2) pseudovirus neutralization assay is needed to facilitate the development of countermeasures. Here, we describe an approach to generate a single-round lentiviral vector-based SARS-CoV-2 pseudovirus, which produced a signal more than 2 logs above background. Specifically, a SARS-CoV-2 spike variant with a cytoplasmic tail deletion of 13 amino acids, termed SΔCT13, conferred enhanced spike incorporation into pseudovirions and increased viral entry into cells compared to that with full-length spike (S). We further compared S and SΔCT13 in terms of their sensitivity to vaccine sera, purified convalescent-phase IgG, human ACE2 (hACE2) murine IgG (mIgG), and the virus entry inhibitor bafilomycin A1 (BafA1). We developed an SΔCT13-based pseudovirus neutralization assay and defined key assay characteristics, including linearity, limit of detection, and intra-assay and intermediate assay precision. Our data demonstrate that the SΔCT13-based pseudovirus shows enhanced infectivity in target cells, which will facilitate the assessment of humoral immunity to SARS-CoV-2 infection, antibody therapeutics, and vaccination. This pseudovirus neutralization assay can also be readily adapted to SARS-CoV-2 variants that emerge.

**IMPORTANCE** Severe acute respiratory syndrome coronavirus 2 (SARS-CoV-2) is the etiologic agent of the COVID-19 pandemic. The development of a high-throughput pseudovirus neutralization assay is critical for the development of vaccines and immune-based therapeutics. In this study, we show that deletion of the cytoplasmic tail of the SARS-CoV-2 spike leads to pseudoviruses with enhanced infectivity. This SΔCT13-based pseudovirus neutralization assay should be broadly useful for the field.

## Abstract (precis)

Severe acute respiratory syndrome coronavirus 2 (SARS-CoV-2) is the etiologic agent of the COVID-19 pandemic. The development of a high-throughput pseudovirus neutralization assay is critical for the development of vaccines and immune-based therapeutics. In this study, we show that deletion of the cytoplasmic tail of the SARS-CoV-2 spike leads to pseudoviruses with enhanced infectivity. This SΔCT13-based pseudovirus neutralization assay should be broadly useful for the field.

## INTRODUCTION

The emerging pandemic of coronavirus disease 2019 (COVID-19), caused by severe acute respiratory syndrome coronavirus 2 (SARS-CoV-2), has led to an unprecedented need to develop vaccines and other countermeasures (1). To develop a successful vaccine candidate, identifying and screening the immune correlates of protection in a high-throughput assay is paramount. Neutralizing antibody responses may represent key immune correlates of protection for SARS-CoV-2 (2, 3). Therefore, it is essential to develop a high-throughput *in vitro* neutralization assay that recapitulates key features of *in vivo* neutralizing activity. However, live SARS-CoV-2 research is restricted to biosafety level 3 (BSL3) facilities, and traditional approaches such as plaque reduction assays are time consuming and low throughput.

Pseudotyped viruses have often been employed to evaluate the physiology of lethal viruses, such as Ebola virus (4), Marburg virus (5), and Lassa virus (6). Pseudoviruses encompass essential components for viral infection, typically, the envelope proteins derived from viruses of interest and an inner core, but they lack an intact viral genome and thus lose self-replication capacity (7). The conformational structure of pseudovirus surface proteins appears to resemble that of the native viral proteins (8), which may allow for a similar viral entry process between the pseudoviruses and infectious counterparts. As such, pseudoviruses are typically regarded as safe and effective virological tools. Pseudoviruses have been utilized for receptor identification, host tropism determination, and viral entry mechanistic studies (7), for identifying entry inhibitors by screening compound libraries (9), and for evaluating neutralizing antibody responses elicited by vaccination or from convalescent-phase sera (10). Pseudoviruses may also be useful in assessments of antibody-dependent enhancement (11, 12). As such, it is important to develop a BSL2-adapted SARS-CoV-2 pseudovirus system.

SARS-CoV-2 encodes four structural proteins, namely, spike (S), nucleocapsid (N), envelope (E), and membrane (M), and more than 20 nonstructural proteins (13). S represents the only protein that mediates SARS-CoV-2 entry and serves as a key immunogen for inducing protective immunity (14). S is synthesized as a precursor in rough endoplasmic reticulum (ER) and further processed into amino-terminal S1 and carboxyl-terminal S2 segments, which contribute to receptor binding and cell fusion, respectively. S has been utilized for generating pseudoviruses for SARS-CoV (SARS-CoV-1) (15) and Middle East respiratory syndrome coronavirus (MERS-CoV) (16), two highly pathogenic and closely related human coronaviruses. As of now, multiple platforms have been leveraged to investigate SARS-CoV-2 entry and/or to determine the induced antibody neutralizing activity, including vesicular stomatitis virus (VSV)-based (17–22), murine leukemia virus (MLV)-based (23–25), and human immunodeficiency virus (HIV)-based pseudotypes (22, 26). In this paper, we observed a rather modest virus infection efficiency with full-length spike protein (S)-based pseudovirus. In contrast, deletion of the last 13 amino acids of the S (SΔCT13) significantly increased spike incorporation into the pseudovirion and improved infectivity. This optimized SΔCT13-based SARS-CoV-2 neutralization assay will be useful in evaluating vaccines and immune-based therapeutics.

## RESULTS

### Deletion of the last 13 amino acids in spike protein enhances SARS-CoV-2 pseudovirus infection.

Two main forms of lentiviral vector-based pseudovirus production strategies have been reported, involving a two-plasmid system or a three-plasmid system (8). The former requires a backbone plasmid that expresses a full set of HIV-1 gene products except for the HIV-1 envelope (Env) and a second plasmid that encodes heterologous Env of interest. In contrast, the three-plasmid system separates the backbone plasmid into two individual constructs, one packaging plasmid and one transfer plasmid (8). The three-plasmid system was generally regarded as a safer method, since multiple virulent genes have been removed (27). For this reason, we chose the three-plasmid system to generate the SARS-CoV-2 pseudovirus, with which we established a pseudovirus-based neutralization assay. The HIV core (backbone) is formulated by the packaging plasmid psPAX2 that expresses the HIV gag and pol proteins and the transfer plasmid pLenti-puro cytomegalovirus (CMV)-Luc that harbors the reporter firefly luciferase gene and essential HIV genomic *cis-*acting regulatory elements allowing proper encapsulation into the HIV capsid (28). During preliminary experiments, the SARS-CoV-2 full-length S demonstrated a rather modest viral entry (2- to 20-fold above cell-only background; data not shown), which limited its utility in evaluating neutralizing activity and thus warranted assay optimization.

**FIG 1:**
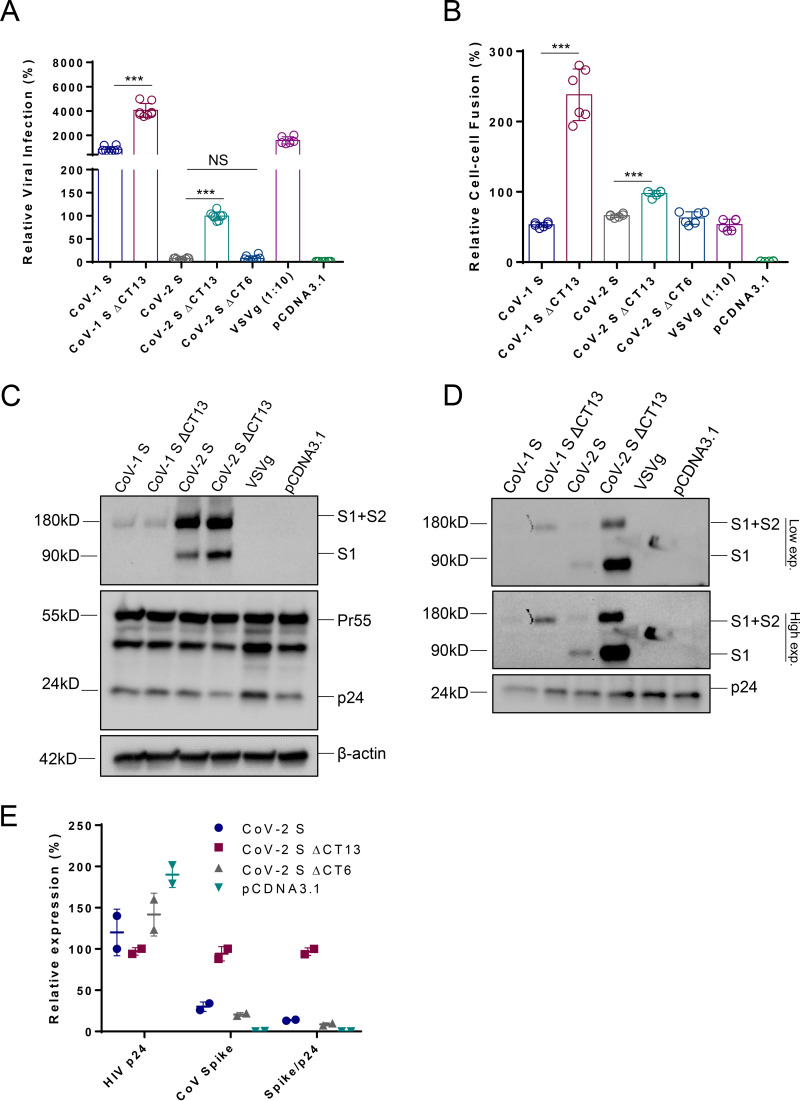
Deletion of the last 13 amino acids in spike protein enhances SARS-CoV-2 pseudovirus infection. (A) Lentiviral particles pseudotyped with CoV-1 S, CoV-1 SΔCT13, CoV-2 S, CoV-2 SΔCT13, CoV-2 SΔCT6, VSVg, or empty (pcDNA3.1) were used to infect HEK293T-hACE2 cells, and the firefly luciferase activity was quantified 48 h postinfection. Data are presented as means ± SDs from 6 to 12 replicates. (B) S, CoV-1 S, CoV-1 SΔCT13, CoV-2 S, CoV-2 SΔCT13, CoV-2 SΔCT6, VSVg, or empty (pcDNA3.1) were cotransfected with an HIV-1 Tat-expressing plasmid into HEK293T cells; meanwhile, pQCXIP-hACE2 was transfected into TZM-bl cells. Cells were mixed in a 1:1 ratio, and the firefly luciferase activity was quantified 12 h postcoculture. Data are presented as means ± SDs from 6 replicates. (C) The cell lysates from virus producer cells in panel A were analyzed by Western blotting. (D) Meanwhile, the viral particles in supernatants were purified, concentrated, and analyzed by Western blotting. Internal control β-actin, HIV-1 p24, and coronavirus spike precursor (S1+S2) and S1 proteins were examined in both cell lysates and viral particles. (E) The concentrations of HIV p24 and SARS-CoV-2 spike of indicated pseudoviruses were assayed by HIV p24 ELISA and SARS-CoV-2 S ELISA. Data from CoV-2 SΔCT13 were set as 100%. The spike density was quantified as Spike/p24. NS, not significant; ***, *P* < 0.001.

Deletion of the last few amino acids in the cytoplasmic tail of SARS-CoV-1 S generated pseudoviruses with greater infectivity (29). We thus sought a similar strategy, deleting the last 13 amino acids in the cytoplasmic tail (CT) of the SARS-CoV-2 S (termed CoV-2 SΔCT13) and evaluating it in HEK293T-human ACE2 (hACE2) cells (Fig. 1A). Interestingly, we noticed significantly increased viral entry for CoV-2 SΔCT13 compared to that for CoV-2 S (Fig. 1A). Similarly, SARS-CoV-1 full-length (CoV-1 S) and the cytoplasmic tail-truncated version (CoV-1 SΔCT13) were included in parallel (Fig. 1A). Both SARS-CoV-1 and SARS-CoV-2 demonstrated augmented virus infection when the last 13 amino acids were ablated, suggesting a possible general mechanism of the enhancement. In particular, SARS-CoV-2 appeared to rely more on CT deletion than SARS-CoV-1, with 24-fold and 2.4-fold increases of infectivity, respectively. The SARS-CoV-1 S showed more than 200-fold enhancement of viral entry compared with that of SARS-CoV-2 S, despite the two sharing the same entry receptor, hACE2. To further explore the determinants of the CoV-2 S, we generated an additional CT variant with the last 6 amino acids deleted (CoV-2 SΔCT6). As shown in Fig. 1A, CoV-2 SΔCT6 displayed similar viral infection efficiency to that of CoV-2 S, suggesting that a potential spike incorporation motif exists in the heptad peptides “SEPVLKG.” We further evaluated the spike protein mediated cell-cell fusion between HEK293T/spike and TZM-bl/hACE2 cells. Deletion of the last 13 amino acids significantly improved CoV-1 S-mediated cell-cell fusion, while CoV-2 SΔCT13 did such to a less extent (Fig. 1B). Consistent with the viral infection result, CoV-2 SΔCT6 induced cell-cell fusion similarly to CoV-2 S (Fig. 1B). To gain mechanistic insight, we performed Western blot analysis of the proteins expressed in virus-producing HEK293T cells. As shown in Fig. 1C, the transfected cells showed a similar expression level of loading control β-actin and HIV core structural protein p24, suggesting comparable transfection efficiency. However, the CoV-1 S plus CoV-2 S (S1+S2) precursors appeared comparable for CoV-1 S, CoV-1 SΔCT13, CoV-2 S, and CoV-2 SΔCT13, whereas S1 expression in CoV-2 SΔCT13 appeared higher than in CoV-2 S, indicating the CT region may affect the spike processing.

We purified viral particles by ultracentrifugation, and analysis by Western blot showed that p24 was generally comparable in all the groups, reflecting similar virus production. However, the S1+S2 and S1 demonstrated a sharp increase (Fig. 1D) for both CoV-1 SΔCT13 and CoV-2 SΔCT13. To confirm, we also collected the virion-containing supernatants and measured the concentration of HIV-1 p24 and CoV-2 spike by enzyme-linked immunosorbent assay (ELISA). In line with Western blot data, more spike proteins as well as a higher spike/p24 ratio were detected in CoV-2 SΔCT13 supernatants (Fig. 1E). The data support a model in which deletion of CT facilitates S incorporation into pseudovirions, thus leading to elevated viral entry efficiency.

**FIG 2:**
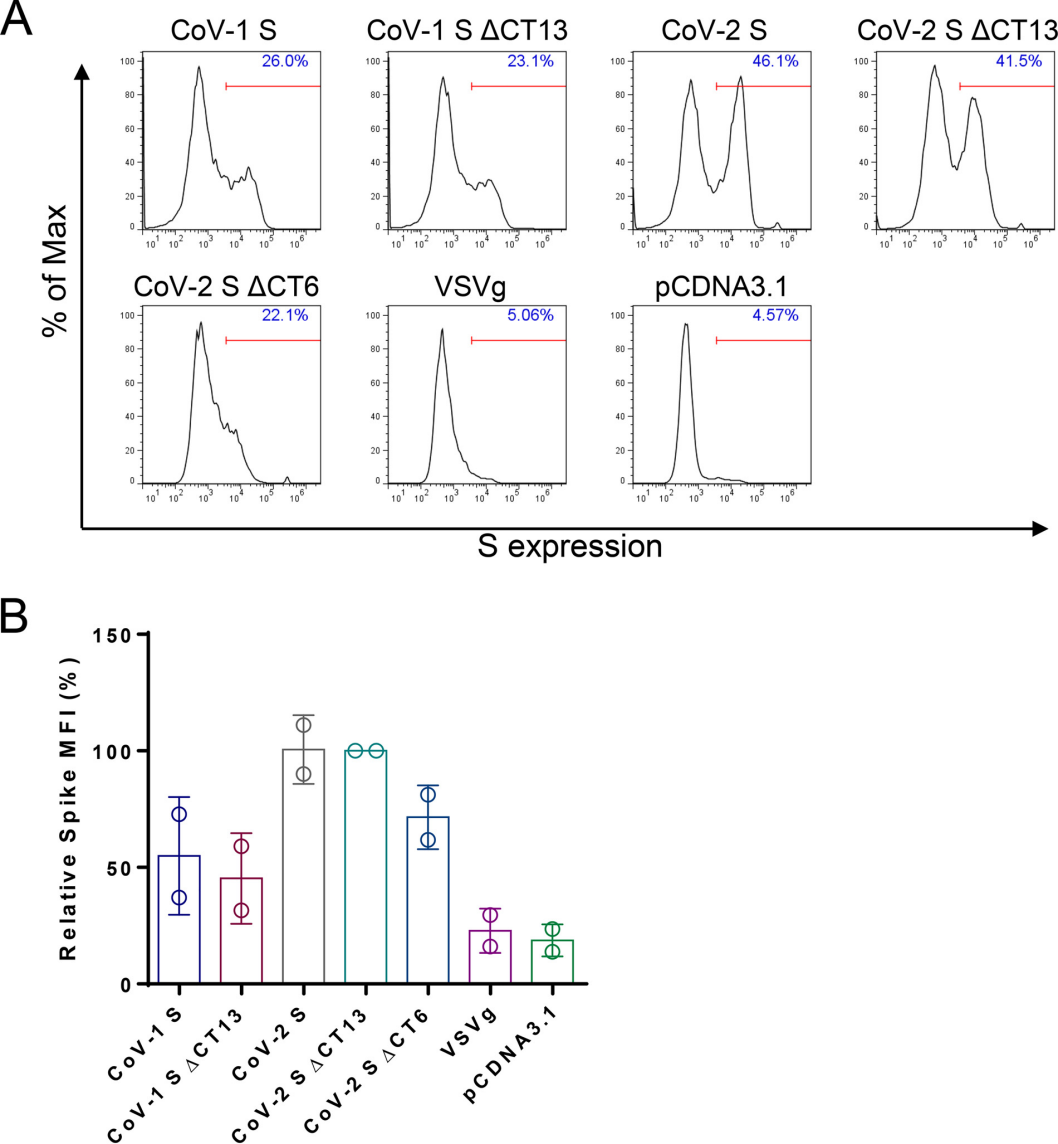
Cell surface staining of SARS-CoV spike expression. The same batch of cells was detached by 5 mM EDTA-DPBS digestion buffer and stained with anti-SARS-CoV-2 RBD antibody. The surface expression of spike proteins was analyzed by flow cytometry. (A) Representative flow plots were analyzed by FlowJo software. (B) Spike expression was quantified as geometric mean fluorescence intensity (MFI). Data are presented as means ± SDs from 2 replicates.

HIV assembles on the plasma membrane, while coronaviruses bud predominantly through ER (30). We tested whether the enhanced S incorporation into pseudovirions with the ΔCT constructs resulted from the higher expression of S on the plasma membrane. We performed cell surface staining of SARS-CoV-1 and SARS-CoV-2 spike proteins. Interestingly, the S and SΔCT13 showed similar expression levels, as reflected by the geometric mean of S intensity (Fig. 2A and B), suggesting that the surface expression level unlikely explains the distinct spike incorporation efficiency. Taken together, the results show that deletion of the last 13 amino acids of S substantially facilitated S incorporation into pseudovirions and viral entry of SARS-CoV-2 in cells. Based on these data, we selected SARS-CoV-2 SΔCT13 for further study.

### SARS-CoV-2 S and SΔCT demonstrated similar sensitivities to neutralization and inhibitor blocking.

**FIG 3:**
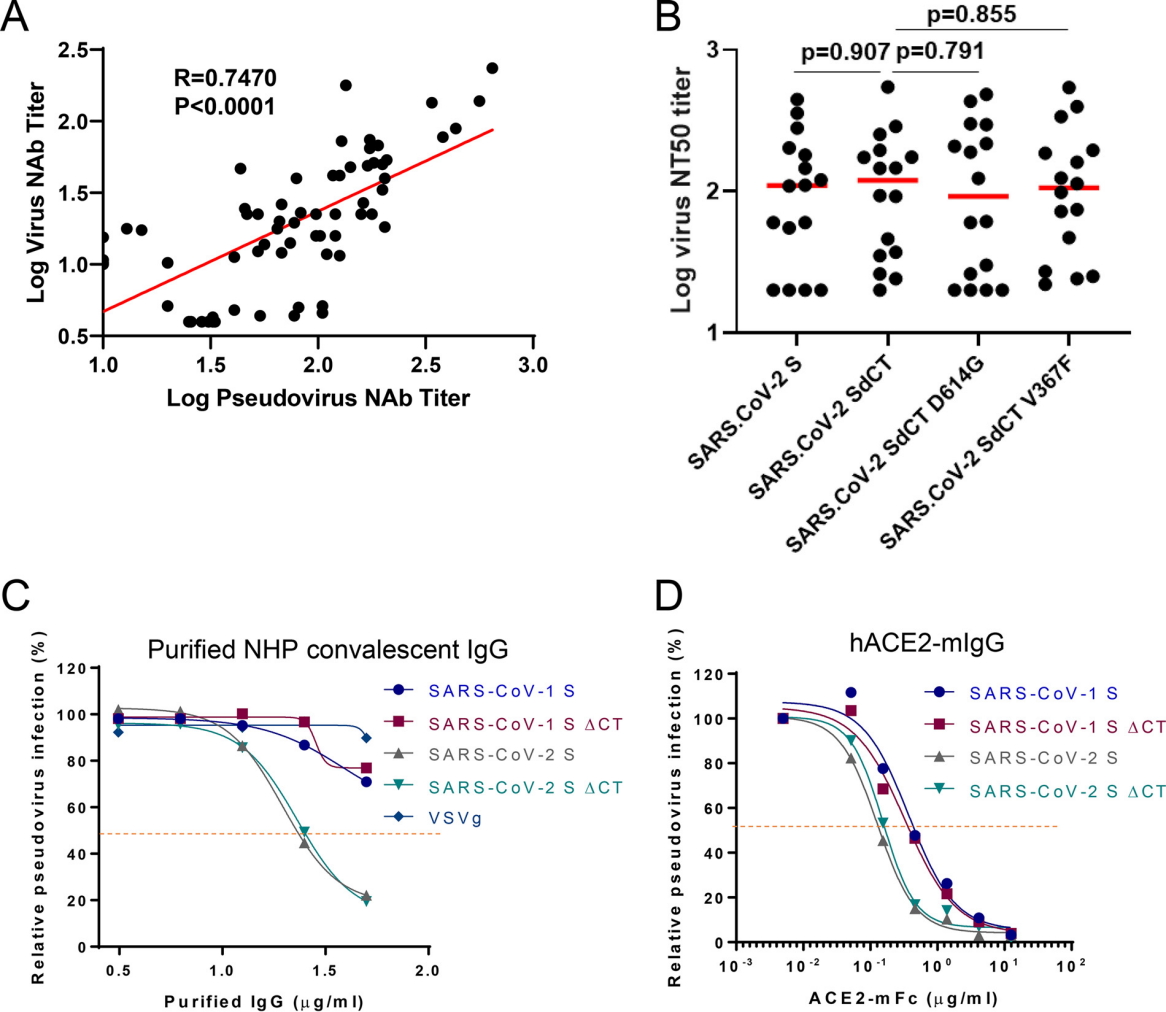
SARS-CoV-2 SΔCT13 maintains the same sensitivity to neutralization and inhibition. (A) Correlation of pseudovirus nAb titers and live virus nAb assays in vaccinated macaques (*N* = 67). Red line reflects the best-fit relationship between these variables. *P* and *R* values reflect two-sided Spearman rank-correlation tests. (B) NT_50_ values of vaccinated macaque serum samples (*N* = 16) against SARS-CoV-2 S, SΔCT, SΔCT D614G and SΔCT V367F pseudovirus. Red bars indicate the median responses. Inhibition curve of pooled purified IgG from convalescent macaques (C) and hACE2-mFc (D) against SARS-CoV-1 S, SARS-CoV-1 SΔCT13, SARS-CoV-2 S, SARS-CoV-2 SΔCT13 or VSVg pseudovirus were generated by preincubating indicated antibody or inhibitors for 1 h and performing virus infection in HEK293T-hACE2 cells. Dotted lines interpolate 50% inhibition or neutralization. Data are presented as means ± standard deviations from technical triplicates.

We next established a neutralization assay with this optimized lentivirus-based pseudovirus (see Materials and Methods for a detailed protocol). To address the question whether the SΔCT13-based pseudovirus neutralization assay would correlate with the live virus neutralization assay, we therefore performed a direct comparison between the pseudovirus-mediated and live virus-mediated neutralization assay results from a total 67 rhesus monkeys and observed a strong correlation (*R* = 0.7470) (Fig. 3A), reflecting that SΔCT13 maintained the key serologic features of the intact/authentic S.

Next, we examined the sensitivity of SARS-CoV-2 S and SARS-CoV-2 SΔCT13 pseudovirus to neutralizing antibodies. To this end, we evaluated immunized rhesus monkey sera (*N* = 16) (Fig. 3B) and purified convalescent-phase IgG from SARS-CoV-2-infected rhesus monkeys (Fig. 3C) toward CoV-2 SΔCT13 or CoV-2 S pseudoviruses. Both the immunized serum and convalescent IgG demonstrated similar neutralization capacity against CoV-2 SΔCT13 and CoV-2 S pseudoviruses (Fig. 3B and C). In particular, we included two additional SARS-CoV-2 S variants, D614G and V367F, which are prevalent virus variants in the global pandemic (31, 32). All groups demonstrated similar 50% neutralization titers (NT_50_ values) (Fig. 3B). Soluble ACE2 also similarly inhibited CoV-1 S, CoV-1 SΔCT13, CoV-2 S, and CoV-2 SΔCT13 pseudoviruses (Fig. 3D). Moreover, these data show the flexibility of this assay in evaluating neutralization of SARS-CoV-2 variants as they emerge.

**FIG 4:**
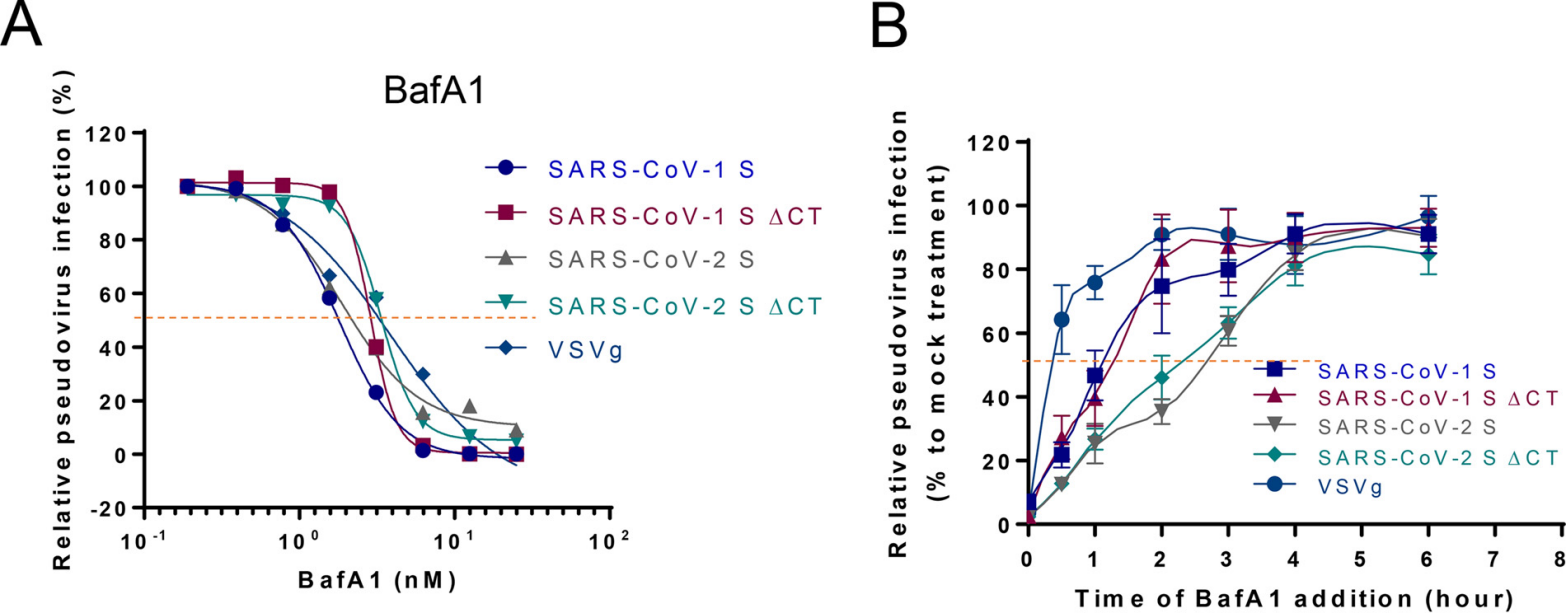
SARS-CoV-2 SΔCT13 maintains the same entry kinetics as the SARS-CoV-2 S. (A) Inhibition curves of BafA1 against CoV-1 S, CoV-1 SΔCT13, CoV-2 S, CoV-2 SΔCT13, or VSVg pseudovirus were generated by preincubating indicated antibody or inhibitors for 1 h and performing virus infection in HEK293T-hACE2 cells. Dotted lines interpolate 50% inhibition or neutralization. (B) The viral entry kinetics of the five pseudoviruses were analyzed by BafA1 block assay. The viruses were bound to target cells at 4°C by spinoculation and then placed at 37°C, allowing virus endocytosis and fusion. The entry inhibitor BafA1 (final concentration 5 nM) was added at indicated time points. Twelve hours posttreatment, the cells were replenished with fresh DMEM. Viral entry was normalized to mock (dimethyl sulfoxide [DMSO]) treated infection group. Data are presented as means ± standard deviations from technical triplicates.

Finally, we measured the entry kinetics of CoV-1 S, CoV-1 SΔCT13, CoV-2 S, CoV-2 SΔCT13 with the inhibitor bafilomycin A1 (BafA1). BafA1 targets the vacuolar-type H^+^-ATPase and disrupts the low-pH environment in intracellular organelles (33), which is essential for entry of several low-pH-dependent viruses, including coronavirus (Fig. 4A). Both SARS-CoV-1 and SARS-CoV-2 demonstrated a comparable half maximal inhibitory concentration (IC_50_) to BafA1 (2 to 5 nM). To characterize the viral entry kinetics, viral entry was initially synchronized by cold arrest (spinoculation at 4°C), which allows efficient virus binding but not virus-cell fusion. BafA1 was added at 0, 0.5, 1, 2, 3, 4, and 6 h post spinoculation at a final concentration of 5 nM, and virus titer was determined 48 h postinfection. As shown in Fig. 4B, the VSVg-mediated viral entry reached plateau around 2 h postinfection, and the half virus entry time (*T*_1/2_) was approximately 25 min, consistent with early reports (34). CoV-1 S and CoV-1 SΔCT13 pseudoviruses demonstrated slower kinetics, requiring 3 h to accomplish maximal viral entry with *T*_1/2_ of approximately 1 h (35). CoV-2 S and CoV-2 SΔCT13 pseudoviruses exhibited even slower entry, with *T*_1/2_ of approximately 2.5 h, suggesting the intrinsic differences between the two coronavirus spikes but no difference between S and SΔCT13 for both viruses. Overall, SARS-CoV-2 S and SΔCT13 demonstrated similar sensitivities to neutralization and inhibition.

### Qualification of the SARS-CoV-2 neutralization assay.

**FIG 5:**
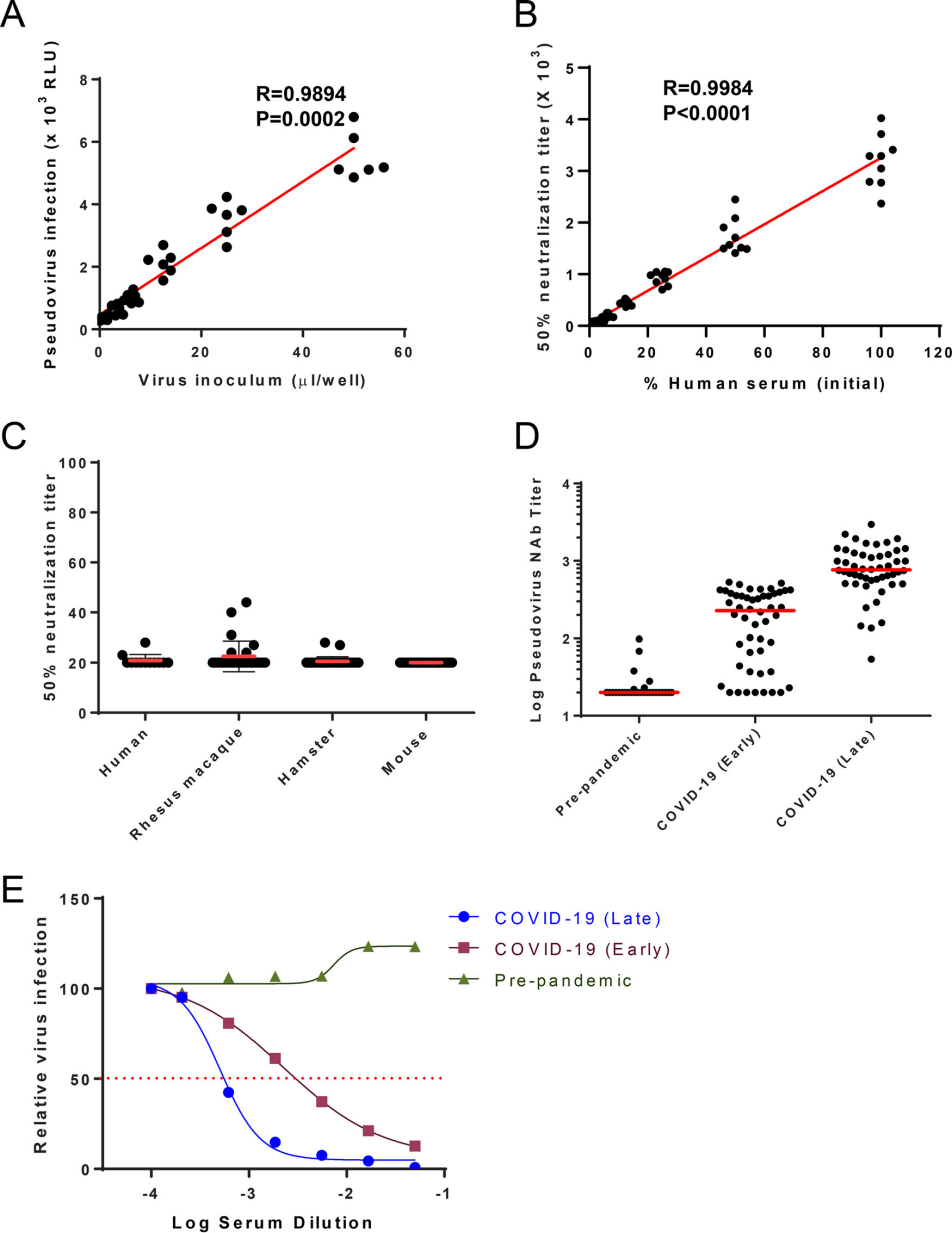
Qualification of the SARS-CoV-2 neutralization assay. (A) The linearity between the virus input volume and firefly luciferase activity was analyzed (N = 5). (B) The linearity between the human serum dilution and NT_50_ was demonstrated (combined data of three operators, with three repeats of each, *N* = 9). *P* and *R* values reflect two-sided Pearson rank-correlation tests. (C) Limit of detection of established neutralization assay was validated by examining serums from COVID-19 prepandemic human donors (*N* = 12), SARS-CoV-2-negative rhesus macaques (*N* = 28), naive hamsters (*N* = 30), and naive BALB/c mice (*N* = 30). Serum samples were 1:20 diluted and then underwent 1:2 serial dilution; data demonstrate 50% neutralization titers. (D) Human serum samples from individuals prepandemic (*n* = 28), with early-stage COVID-19 (within 10 days since symptom onset, *n* = 50), and with late-stage COVID-19 (greater than 10 days since symptom onset, *n* = 50) were applied to an established SARS-CoV-2 neutralization assay. Data demonstrate 50% neutralization titers, and red bars reflect the median titers. (E) Representative data from each group were plotted as inhibition curves.

As qualification experiments, we assessed the following parameters: linearity, limit of detection (LOD), specificity, and reproducibility. Linearity between input virus and luciferase activity was analyzed with increasing amounts of pseudovirus. As shown in Fig. 5A, solid linearity was achieved between 3.125- and 50-μl virus inoculums (*R* = 0.9894, *P* < 0.0001). Of note, 1 μl of input virus was equivalent to 4 pg of HIV-1 p24, as determined by HIV-1 p24 ELISA. To determine sample dilutional linearity combined with intermediate precision, a pooled, heat-inactivated, high-titer COVID-19 human convalescent-phase serum sample was tested undiluted (neat) and in 2-fold serial dilutions ranging from 1:1 to 1:32. These assays were performed by three different operators over three different days. Linear regression of NT_50_ values plotted as a function of the serum dilution factor suggested strong linearity when the serum concentration was between undiluted and 1:16 dilution (*R* = 0.9984, *P* < 0.0001) (Fig. 5B).

Intra-assay and intermediate assay precision was assessed using the dilutional linearity sample data. Using prespecified acceptance criteria of ≤25% coefficient of variation (%CV) to define the upper limit of quantification (ULOQ), the assay ULOQ was established at an NT_50_ of 3,153 (Table 1). The limit of detection (LOD) was assessed by measuring the neutralizing activity of human serum starting with a dilution of 1:20. Sera from three other model species, rhesus macaque, hamster, and mouse (naive serum), were included for comparison. The LOD was established to 1:23, 1:28, 1:22, and 1:20 for human, rhesus macaque, hamster, and mouse, respectively (Fig. 5C). Due to limited availability of high-volume COVID-19 human convalescent-phase serum samples at the time of assay development, undiluted and 1:16 diluted samples were used to approximate intra-assay precision at the high and low ends of the assay range with samples meeting predefined criteria of CV of ≤25% at each level for each operator (Table 2). Development of discrete quality control samples that fall in the high (∼80%), medium (∼50%), and low (∼20%) ranges for established assay range will subsequently be developed as high-volume high-neutralizing antibody (nAb)-titer reagents become available. The lentivirus-based neutralization assay demonstrated an overall assay precision of 15.7%.

**TABLE 1:** Intermediate assay precision with pooled convalescent human serum

Serum dilution	Precision											
	Operator 1			Operator 2			Operator 3			Geometricmean	SD	%CV
	Run 1	Run 2	Run 3	Run 1	Run 2	Run 3	Run 1	Run 2	Run 3			
Undiluted	2,370	3,290	3,411	3,047	2,788	3,714	4,024	2,774	3,289	3,153.1	508.2	16.1
1:1	1,488	1,410	1,710	1,567	1,498	1,907	2,447	1,516	2,086	1,708.5	346.3	20.3
1:2	763	912	983	704	846	983	1,039	1,042	1,054	916.4	128.4	14.0
1:4	391	464	445	364	527	481	462	503	434	449.6	51.3	11.4
1:8	193	210	166	168	224	254	251	168	177	198.3	35.4	17.8
1:16	111	101	111	75	97	99	98	83	87	95.1	12.2	12.8
1:32	46	28	21	31	43	38	52	26	31	33.9	10.2	30.0

**TABLE 2:** Intra-assay precision with pooled convalescent human serum

Serum dilution	NT_50_					
	Run 1	Run 2	Run 3	Geometric mean	SD	%CV
Operator 1
Undiluted	2,370	3,290	3,411	2,985	569.3	19.1
1:16	111	101	111	108	5.8	5.4
Operator 2
Undiluted	3,047	2,788	3,714	3,160	477.7	15.1
1:16	75	97	99	90	13.3	14.9
Operator 3
Undiluted	4,024	2,774	3,289	3,324	628.2	18.9
1:16	98	83	87	89	7.8	8.7

With this established SARS-CoV-2 neutralization assay, we tested human serum from individuals prepandemic (*n* = 28), at an early stage of COVID-19 (within 10 days since symptom onset, *n* = 50), and at a late stage of COVID-19 (greater than 10 days since symptom onset, *n* = 50). There was a clear enhanced neutralizing antibody titer upon infection, with median titers of 20, 218, and 776, respectively (Fig. 5D and E).

## DISCUSSION

Pseudoviruses are versatile and valuable tools for both basic and applied virologic research and have particular advantages when live virus requires higher biosafety BSL3 or BSL4 facilities. High-throughput assays using pseudoviruses in BSL2 facilities can facilitate rapid testing and development of SARS-CoV-2 countermeasures such as vaccines. As such, SARS-CoV-2 pseudovirus neutralization assays are being established. In the manuscript, we describe the development and qualification of an optimized SARS-CoV-2 pseudovirus neutralization assay for preclinical studies and clinical trials. We found that the full-length S of two closely related human coronaviruses, SARS-CoV-1 and MERS-CoV, were readily translatable into a standard pseudovirus system (15, 16). Full-length SARS-CoV-2 S mediated a low level of infection, but this can be improved by deleting 13 amino acids in the CT.

We addressed several key issues in the development of this assay. First, expression of the virus-derived coding sequence in mammalian cells may be suboptimal due to potential codon usage bias (36). We therefore codon optimized the entire spike DNA sequence by replacing the GC stretches with Homo sapiens-biased codons and validated its robust expression profile in HEK293T cells (Fig. 1C). Second, the viral entry efficiency of SARS-CoV-2 into target cells appears to be intrinsically inferior to that of SARS-CoV-1. SARS-CoV-1 exhibited a >100-fold higher entry efficiency than SARS-CoV-2 (Fig. 1A). This may be ascribed to the stronger binding affinity to the receptor hACE2 (37), greater spike incorporation, accelerated entry kinetics, or other factors. Regardless, we sought to improve SARS-CoV-2 pseudovirus entry into target cells to improve the assay performance. Because partial deletion of the SARS-CoV-1 CT was reported to increase the viral infectivity (29), we deleted the last 13 amino acids of the cytoplasmic tail for both SARS-CoV-1 and SARS-CoV-2. Both SARS-CoVs demonstrated greater viral entry, but this effect was more pronounced for SARS-CoV-2 (Fig. 1A), with a 20-fold enhancement (22). We also observed a marked increase in SΔCT incorporation into pseudovirions compared to that of S for both SARS-CoV-1 and SARS-CoV-2, which correlated with enhanced viral entry. The observed SARS-CoV S protein expressed on the cell surface (Fig. 2) may be due to leakiness of the ER retention upon overexpression (29). We hypothesize that the enhanced incorporation of SΔCT may reflect higher cell surface expression as a result of the removal of a reported ER retention motif KxHxx in the last 13 amino acids (26, 38). However, we observed the same cell surface expression levels for both SΔCT13 and S. In addition, the SΔCT6 deleted the KxHXX motif but maintained a similar viral entry profile to that of the S, arguing against the hypothesis that the spike cell surface expression makes a difference. Interestingly, when we performed a cell-cell fusion assay, where the CoV-2 S and CoV-2 SΔCT13 had comparable expression levels (Fig. 1C and 2), cell-cell fusion events were similarly induced (<2-fold difference) (Fig. 1B), suggesting that the functioning of the spike may not be altered by CT deletion. A direct measurement of viral entry kinetics (Fig. 4B) similarly implicated that truncation of the 13 amino acids unlikely altered the viral entry properties.

One question regarding the use of SΔCT is whether the C-terminal deletion may have changed the conformation of the protein, thus potentially altering neutralization sensitivity. There is currently a lack of direct structural evidence for the CT region of SARS-CoV-2 (39). We observed a strong positive correlation between the pseudovirus- and live virus-based neutralization assays (Fig. 3A), suggesting the utility of the pseudovirus neutralization assay. Interestingly, we noticed that a subset of samples with modest NT_50_ values were detected with the pseudovirus assay but not with the live virus assay, suggesting different assay sensitivities. Additional studies are warranted to test this hypothesis. Moreover, immunologic and virologic comparisons between SARS-CoV-2 pseudoviruses with S and SΔCT13 suggested similar sensitivities to polyclonal antibody, hACE2-mIgG, and BafA1. Therefore, it is probably quantitative rather than qualitative changes of the spike in the virion that determine the increased viral infection rate. Further investigation including detailed structural insight may be warranted. Currently, VSV-based and lentiviral vector-based pseudovirus systems stand for the primary assays for neutralizing antibody titer determination (17–22, 26). The intrinsic properties of rapid replication of infectious VSV accelerate the data generation, while the lentiviral vectors morphologically resemble SARS-CoV-2 and may more genuinely reflect the entry process of SARS-CoV-2.

To define performance characteristics of this SARS-CoV-2 pseudovirus neutralization assay, we performed a series of qualification experiments. The limits of detection for four species appear to be similar (approximately 1:20). Intra- and intermediate precision demonstrated precision levels of <25%. Overall, this optimized and qualified assay has been developed to evaluate SARS-CoV-2 neutralizing antibody responses in both preclinical and clinical studies. Moreover, this pseudovirus neutralization assay is readily adaptable to incorporating S sequences from SARS-CoV-2 variants, such as currently circulating UK and South African variants.

## MATERIALS AND METHODS

### Plasmids, cells, and reagents.

Sequences of full-length spike (S) or 13 amino acids in cytoplasmic tail deleted S (SΔCT) of SARS-CoV-1 (Tor2, GenBank accession AAP41037.1) and SARS-CoV-2 (Wuhan/WIV04/2019) were codon optimized and commercially synthesized (Integrated DNA Technologies, NJ, USA). Synthetic genes were cloned into the mammalian expression plasmid pcDNA3.1+ (Invitrogen, CA, USA). SARS-CoV-2 S D614G and V367F variants were generated by site-directed mutagenesis based on SARS-CoV-2 SΔCT. Human ACE2 genes were commercially synthesized (Integrated DNA Technologies, NJ, USA) and further cloned into pQCXIP retroviral vector (catalog number [cat. no.] 631516; TaKaRa). pHEF-VSVG expressing vesicular stomatitis virus (VSV) glycoprotein was obtained through the NIH AIDS Reagent Program, Division of AIDS, NIAID, NIH (cat. no. 4693). PsPAX2 (cat. no. 12260), pLenti-CMV-Puro-Luc (cat. no. 17447), and pBS-CMV-gag-pol (cat. no. 35614) were obtained from Addgene (Boston, MA).

HEK293T (ATCC CRL-11268), Vero-E6 (ATCC CRL-1586) and 786-O (ATCC CRL-1932), and TZM-bl (AIDS Reagent Program, cat. no. 8129) cells were maintained in Dulbecco’s modified Eagle’s medium (DMEM) supplemented with 0.5% penicillin-streptomycin and 5% fetal bovine serum (FBS). HEK293T-hACE2 cells were maintained in DMEM supplemented with 0.5% penicillin-streptomycin, 5% FBS, and 1 μg/ml puromycin (Sigma).

Rabbit polyclonal antibody against SARS-CoV-2 receptor-binding domain (RBD) and hACE2 conjugated with mouse IgG Fc (hACE2-mFc; cat. no. 10108) were purchased from Sino Biological. Bafilomycin A1 was purchased from Sigma. Horseradish peroxidase (HRP)-conjugated beta actin antibody (cat. no. HRP-60008) was purchased from Proteintech. HIV p24 ELISA kit was ordered from Abcam (cat. no. ab218268).

### Rhesus and human serum samples.

Rhesus macaque anti-SARS-CoV-2 serum was obtained after an adenoviral vector-based vaccination (3). Serum samples from 16 animals were collected 4 weeks post-single-shot vaccination. The convalescent-phase serum IgG from our early study (40) was purified and pooled. Twelve deidentified SARS-CoV-2 prepandemic human serum samples from Boston, MA, were obtained. All human studies were conducted in compliance with all relevant local, state, and federal regulations and were approved by the Partners Institutional Review Board (IRB).

### Generation of HEK293T-hACE2 stable cells.

The retroviral pseudotypes expressing human ACE2 (hACE2) were generated by cotransfecting HEK293T cells with pHEF-VSVG, pBS-CMV-gag-pol, and pQCXIP-hACE2 at a ratio of 0.5:1:1. Viruses in the supernatants were collected every 12 h. The collected viruses were used to transduce HEK293T cells, and positive cell populations were selected by using 1 μg/ml puromycin.

### Production of pseudotyped lentiviral particles.

The SARS-CoV-2 pseudoviruses expressing a luciferase reporter gene were generated in an approach similar to that described previously (2, 40, 41). Ten micrograms packaging construct psPAX2, 10 μg luciferase reporter plasmid pLenti-CMV Puro-Luc, and 5 μg spike protein expressing pcDNA3.1-SARS CoV-2 SΔCT were cotransfected into 5 × 10^6^ HEK293T cells in a 10-cm dish with lipofectamine 2000 (Sigma). Six hours posttransfection, the supernatants were replaced with fresh DMEM (plus 5% FBS). The supernatants containing the pseudotype viruses were collected 48 h posttransfection; pseudotype viruses were purified by filtration with a 0.45-μm filter.

### Lentiviral luciferase-based neutralization assay.

The SARS-CoV-2 pseudoviruses neutralization assay was generated with an approach similar to that described previously (2, 40, 41). To determine the neutralization activity of the serum, plasma, or IgG samples from cohorts, HEK293T-hACE2 cells were seeded in 96-well tissue culture plates at a density of 1.75 × 10^4^ cells/well overnight. Threefold serial dilutions of heat-inactivated plasma samples were prepared and mixed with 50 μl of pseudovirus. The mixture was incubated at 37°C for 1 h before adding to HEK293T-hACE2 cells. Forty-eight hours after infection, cells were lysed in Steady-Glo luciferase assay (Promega) according to the manufacturer’s instructions. SARS-CoV-2 neutralization titers were defined as the sample dilution at which a 50% reduction in relative light unit (RLU) was observed relative to the average from the virus control wells.

### Live virus neutralization assay.

A full-length SARS-CoV-2 virus based on a Seattle, WA, isolate was designed to express luciferase and green fluorescence protein (GFP) and was recovered via reverse genetics. The titer of the virus was determined in Vero E6 USAMRID cells to obtain a relative light units (RLU) signal of at least 10× the cell-only control background. Vero E6 USAMRID cells were plated at 20,000 cells per well the day prior in clear-bottom black-walled 96-well plates. Neutralizing antibody serum samples were tested at a starting dilution of 1:40 and were serially diluted 4-fold up to eight dilution spots. Antibody-virus complexes were incubated at 37°C with 5% CO_2_ for 1 h. Following incubation, growth medium was removed, and virus-antibody dilution complexes were added to the cells in duplicates. Virus-only controls and cell-only controls were included in each neutralization assay plate. Following infection, plates were incubated at 37°C with 5% CO_2_ for 48 h. After the 48 h of incubation, cells were lysed, and luciferase activity was measured via a Nano-Glo luciferase assay system (Promega) according to the manufacturer’s specifications. SARS-CoV-2 neutralization titers were defined as the sample dilution at which a 50% reduction in RLU was observed relative to the average from the virus control wells.

### Cell-cell fusion assay.

HEK293T cells were transfected with spike-encoding plasmids along with HIV-1 Tat optimized expression vector (AIDS Reagent Program, cat. no. 827). Meanwhile, TZM-bl cells were transfected with human ACE2 plasmid. Twenty-four hours after transfection, cells were cocultured with TZM-bl cells at 1:1 ratio for 12 h, lysed, and measured for firefly luciferase activity. All samples were tested in duplicates, and the results were averaged.

### Virus entry kinetics.

Briefly, 1.5 × 10^4^ HEK293T-ACE2 cells/well were seeded in a flat 96-well plate at a density of 1.5 × 10^5^ cells per ml. The second day, 50 μl virus was added into each well, and the plate was spun at 1,680 × *g* for 30 min at 4°C. The cell culture plates were transferred into a 37°C incubator immediately after the spin to initiate viral internalization and infection. Fifteen microliters DMEM containing 50 nM BafA1 was added into each well immediately after spinoculation (0 h) and 0.5, 1, 2, 3, 4, and 6 h post-temperature shift. Twelve hours after culturing, cells were replenished with fresh DMEM. Forty-eight hours postspinoculation, cells were lysed for luciferase activity quantification.

### Western blotting.

Western blotting was performed as previously described (42). Briefly, transfected HEK293T cells were collected, washed once with icy 1× Dulbecco’s phosphate-buffered saline (DPBS; Sigma), and lysed in radioimmunoprecipitation assay buffer (RIPA; Thermo Fisher, MA) for 20 min on ice. The pseudoviral particles were purified from DMEM culture medium by ultracentrifugation (32,000 rpm, 2 h, 4°C) and resuspended in 1× DPBS. The cell lysates and viral particles were dissolved in sample buffer (4×; Thermo Fisher, MA), separated on a 4% to 15% gradient gel (Bio-Rad), and detected by anti-HIV-1 p24, anti-SARS-CoV-2 RBD, and anti-β-actin antibodies.

### Cell surface staining.

Cells were washed twice with cold 1× DPBS plus 2% FBS, detached with 1× DPBS containing 5 mM EDTA, and incubated on ice with the appropriate primary antibodies for 1 h. After three washes with PBS plus 2% FBS, cells were further incubated with fluorescein isothiocyanate (FITC)-conjugated secondary antibodies for 45 min. After two washes, cells were fixed with 2% formaldehyde and analyzed in a BD LSRII flow cytometer.

### Intermediate precision and dilutional linearity.

A pooled, heat-inactivated high-titer COVID-19 convalescent-phase serum was undiluted or serially prediluted in 2-fold dilutions (i.e., neat, 1:1, 1:2, 1:4, 1:8, 1:16, and 1:32). Each of these diluted samples was measured with a starting dilution of 1:20 and further 1:2 serial dilution. These assays were performed by three independent operators over three different days.

### Intra-assay precision.

In the absence of predefined, precalibrated quality control samples, two dilutions within the linear range established in the intermediate precision and dilutional linearity assay were selected to approximate high and low levels of the range. These values subsequently were used to determine intra-assay precision.

### Limit of detection.

The LOD was determined by testing multiple COVID-19 prepandemic negative serums. To establish the LOD, 12 prepandemic negative human serum samples, 28 naive rhesus monkey serum samples, 30 naive hamster serum samples, and 30 naive mouse serum samples were utilized to set the LOD. The LOD of the assay was determined as the mean of the negative serum samples plus the standard deviation (SD) on the assay nominal scale.

### Statistical analyses.

All statistical analyses were carried out in GraphPad Prism 8 with Student’s *t* tests or one-way analysis of variance unless otherwise noted. Typically, data from at least three independent experiments were used for analyses.

## References

[1] Corey L, Mascola JR, Fauci AS, Collins FS. 2020. A strategic approach to COVID-19 vaccine R&D. Science 368:948–950. 10.1126/science.abc5312. 32393526

[2] Yu J, Tostanoski LH, Peter L, Mercado NB, McMahan K, Mahrokhian SH, Nkolola JP, Liu J, Li Z, Chandrashekar A, Martinez DR, Loos C, Atyeo C, Fischinger S, Burke JS, Slein MD, Chen Y, Zuiani A, Lelis FJN, Travers M, Habibi S, Pessaint L, Van Ry A, Blade K, Brown R, Cook A, Finneyfrock B, Dodson A, Teow E, Velasco J, Zahn R, Wegmann F, Bondzie EA, Dagotto G, Gebre MS, He X, Jacob-Dolan C, Kirilova M, Kordana N, Lin Z, Maxfield LF, Nampanya F, Nityanandam R, Ventura JD, Wan H, Cai Y, Chen B, Schmidt AG, Wesemann DR, Baric RS, et al. 2020. DNA vaccine protection against SARS-CoV-2 in rhesus macaques. Science 369:806–811. 10.1126/science.abc6284. 32434945 PMC7243363

[3] Mercado NB, Zahn R, Wegmann F, Loos C, Chandrashekar A, Yu J, Liu J, Peter L, McMahan K, Tostanoski LH, He X, Martinez DR, Rutten L, Bos R, van Manen D, Vellinga J, Custers J, Langedijk JP, Kwaks T, Bakkers MJG, Zuijdgeest D, Huber SKR, Atyeo C, Fischinger S, Burke JS, Feldman J, Hauser BM, Caradonna TM, Bondzie EA, Dagotto G, Gebre MS, Hoffman E, Jacob-Dolan C, Kirilova M, Li Z, Lin Z, Mahrokhian SH, Maxfield LF, Nampanya F, Nityanandam R, Nkolola JP, Patel S, Ventura JD, Verrington K, Wan H, Pessaint L, Ry AV, Blade K, Strasbaugh A, Cabus M, Brown R, Cook A, et al. 2020. Single-shot Ad26 vaccine protects against SARS-CoV-2 in rhesus macaques. Nature 586:583–588. 10.1038/s41586-020-2607-z. 32731257 PMC7581548

[4] Salata C, Calistri A, Alvisi G, Celestino M, Parolin C, Palu G. 2019. Ebola virus entry: from molecular characterization to drug discovery. Viruses 11:274. 10.3390/v11030274. PMC6466262 30893774

[5] Chen Q, Tang K, Zhang X, Chen P, Guo Y. 2018. Establishment of pseudovirus infection mouse models for *in vivo* pharmacodynamics evaluation of filovirus entry inhibitors. Acta Pharm Sin B 8:200–208. 10.1016/j.apsb.2017.08.003. 29719780 PMC5925413

[6] Li Q, Liu Q, Huang W, Wu J, Nie J, Wang M, Zhao C, Zhang L, Wang Y. 2017. An LASV GPC pseudotyped virus based reporter system enables evaluation of vaccines in mice under non-BSL-4 conditions. Vaccine 35:5172–5178. 10.1016/j.vaccine.2017.07.101. 28797730

[7] Sanders DA. 2002. No false start for novel pseudotyped vectors. Curr Opin Biotechnol 13:437–442. 10.1016/s0958-1669(02)00374-9. 12459334

[8] Li Q, Liu Q, Huang W, Li X, Wang Y. 2018. Current status on the development of pseudoviruses for enveloped viruses. Rev Med Virol 28:e1963. 10.1002/rmv.1963. PMC7169153 29218769

[9] Basu A, Mills DM, Bowlin TL. 2010. High-throughput screening of viral entry inhibitors using pseudotyped virus. Curr Protoc Pharmacol Chapter 13:Unit 13B.3. 10.1002/0471141755.ph13b03s51. 21935898

[10] Walker LM, Huber M, Doores KJ, Falkowska E, Pejchal R, Julien JP, Wang SK, Ramos A, Chan-Hui PY, Moyle M, Mitcham JL, Hammond PW, Olsen OA, Phung P, Fling S, Wong CH, Phogat S, Wrin T, Simek MD, Protocol G Principal Investigators, Koff WC, Wilson IA, Burton DR, Poignard P. 2011. Broad neutralization coverage of HIV by multiple highly potent antibodies. Nature 477:466–470. 10.1038/nature10373. 21849977 PMC3393110

[11] Wan Y, Shang J, Sun S, Tai W, Chen J, Geng Q, He L, Chen Y, Wu J, Shi Z, Zhou Y, Du L, Li F. 2019. Molecular mechanism for antibody-dependent enhancement of coronavirus entry. J Virol 94:e02015-19. 10.1128/JVI.02015-19. PMC7022351 31826992

[12] Kushnir N, Streatfield SJ, Yusibov V. 2012. Virus-like particles as a highly efficient vaccine platform: diversity of targets and production systems and advances in clinical development. Vaccine 31:58–83. 10.1016/j.vaccine.2012.10.083. 23142589 PMC7115575

[13] Kim D, Lee JY, Yang JS, Kim JW, Kim VN, Chang H. 2020. The architecture of SARS-CoV-2 transcriptome. Cell 181:914.e10–921.e10. 10.1016/j.cell.2020.04.011. 32330414 PMC7179501

[14] Du L, He Y, Zhou Y, Liu S, Zheng BJ, Jiang S. 2009. The spike protein of SARS-CoV–a target for vaccine and therapeutic development. Nat Rev Microbiol 7:226–236. 10.1038/nrmicro2090. 19198616 PMC2750777

[15] Han DP, Kim HG, Kim YB, Poon LL, Cho MW. 2004. Development of a safe neutralization assay for SARS-CoV and characterization of S-glycoprotein. Virology 326:140–149. 10.1016/j.virol.2004.05.017. 15262502 PMC7127165

[16] Zhao G, Du L, Ma C, Li Y, Li L, Poon VK, Wang L, Yu F, Zheng BJ, Jiang S, Zhou Y. 2013. A safe and convenient pseudovirus-based inhibition assay to detect neutralizing antibodies and screen for viral entry inhibitors against the novel human coronavirus MERS-CoV. Virol J 10:266. 10.1186/1743-422X-10-266. 23978242 PMC3765664

[17] Nie J, Li Q, Wu J, Zhao C, Hao H, Liu H, Zhang L, Nie L, Qin H, Wang M, Lu Q, Li X, Sun Q, Liu J, Fan C, Huang W, Xu M, Wang Y. 2020. Establishment and validation of a pseudovirus neutralization assay for SARS-CoV-2. Emerg Microbes Infect 9:680–686. 10.1080/22221751.2020.1743767. 32207377 PMC7144318

[18] Case JB, Rothlauf PW, Chen RE, Liu Z, Zhao H, Kim AS, Bloyet LM, Zeng Q, Tahan S, Droit L, Ilagan MXG, Tartell MA, Amarasinghe G, Henderson JP, Miersch S, Ustav M, Sidhu S, Virgin HW, Wang D, Ding S, Corti D, Theel ES, Fremont DH, Diamond MS, Whelan SPJ. 2020. Neutralizing antibody and soluble ACE2 inhibition of a replication-competent VSV-SARS-CoV-2 and a clinical isolate of SARS-CoV-2. Cell Host Microbe 28:475.e5–485.e5. 10.1016/j.chom.2020.06.021. 32735849 PMC7332453

[19] Letko M, Munster V. 22 1 2020. Functional assessment of cell entry and receptor usage for lineage B beta-coronaviruses, including 2019-nCoV. bioRxiv 10.1101/2020.01.22.915660. PMC7095430 32094589

[20] Xiong H-L, Wu Y-T, Cao J-L, Yang R, Ma J, Qiao X-Y, Yao X-Y, Zhang B-H, Zhang Y-L, Hou W-H, Shi Y-, Xu J-J, -Zhang L, Wang S-J, Fu B-R, Yang T, Ge S-X, Zhang J, Yuan Q, Huang B-Y, Li Z-Y, Zhang T-Y, Xia N-S. 2020. Robust neutralization assay based on SARS-CoV-2 S-bearing vesicular stomatitis virus (VSV) pseudovirus and ACE2-overexpressed BHK21 cells. Emerg Microbes Infect 9:2105–2113. 10.1080/22221751.2020.1815589. 32893735 PMC7534347

[21] Hoffmann M, Kleine-Weber H, Schroeder S, Kruger N, Herrler T, Erichsen S, Schiergens TS, Herrler G, Wu NH, Nitsche A, Muller MA, Drosten C, Pohlmann S. 2020. SARS-CoV-2 cell entry depends on ACE2 and TMPRSS2 and is blocked by a clinically proven protease inhibitor. Cell 181:271.e8–280.e8. 10.1016/j.cell.2020.02.052. 32142651 PMC7102627

[22] Schmidt F, Weisblum Y, Muecksch F, Hoffmann H-H, Michailidis E, Lorenzi JCC, Mendoza P, Rutkowska M, Bednarski E, Gaebler C, Agudelo M, Cho A, Wang Z, Gazumyan A, Cipolla M, Caskey M, Robbiani DF, Nussenzweig MC, Rice CM, Hatziioannou T, Bieniasz PD. 2020. Measuring SARS-CoV-2 neutralizing antibody activity using pseudotyped and chimeric viruses. J Exp Med 217:e20201181. 10.1084/jem.20201181. 32692348 PMC7372514

[23] Walls AC, Park YJ, Tortorici MA, Wall A, McGuire AT, Veesler D. 2020. Structure, function, and antigenicity of the SARS-CoV-2 spike glycoprotein. Cell 181:281.e6–292.e6. 10.1016/j.cell.2020.02.058. 32155444 PMC7102599

[24] Pinto D, Park YJ, Beltramello M, Walls AC, Tortorici MA, Bianchi S, Jaconi S, Culap K, Zatta F, De Marco A, Peter A, Guarino B, Spreafico R, Cameroni E, Case JB, Chen RE, Havenar-Daughton C, Snell G, Telenti A, Virgin HW, Lanzavecchia A, Diamond MS, Fink K, Veesler D, Corti D. 2020. Cross-neutralization of SARS-CoV-2 by a human monoclonal SARS-CoV antibody. Nature 583:290–295. 10.1038/s41586-020-2349-y. 32422645

[25] Pinto D, Park YJ, Beltramello M, Walls AC, Tortorici MA, Bianchi S, Jaconi S, Culap K, Zatta F, De Marco A, Peter A, Guarino B, Spreafico R, Cameroni E, Case JB, Chen RE, Havenar-Daughton C, Snell G, Telenti A, Virgin HW, Lanzavecchia A, Diamond MS, Fink K, Veesler D, Corti D. 9 4 2020. Structural and functional analysis of a potent sarbecovirus neutralizing antibody. bioRxiv 10.1101/2020.04.07.023903.

[26] Crawford KHD, Eguia R, Dingens AS, Loes AN, Malone KD, Wolf CR, Chu HY, Tortorici MA, Veesler D, Murphy M, Pettie D, King NP, Balazs AB, Bloom JD. 2020. Protocol and reagents for pseudotyping lentiviral particles with SARS-CoV-2 spike protein for neutralization assays. Viruses 12:513. 10.3390/v12050513. PMC7291041 32384820

[27] Dull T, Zufferey R, Kelly M, Mandel RJ, Nguyen M, Trono D, Naldini L. 1998. A third-generation lentivirus vector with a conditional packaging system. J Virol 72:8463–8471. 10.1128/JVI.72.11.8463-8471.1998. 9765382 PMC110254

[28] Campeau E, Ruhl VE, Rodier F, Smith CL, Rahmberg BL, Fuss JO, Campisi J, Yaswen P, Cooper PK, Kaufman PD. 2009. A versatile viral system for expression and depletion of proteins in mammalian cells. PLoS One 4:e6529. 10.1371/journal.pone.0006529. 19657394 PMC2717805

[29] Giroglou T, Cinatl J, Jr, Rabenau H, Drosten C, Schwalbe H, Doerr HW, von Laer D. 2004. Retroviral vectors pseudotyped with severe acute respiratory syndrome coronavirus S protein. J Virol 78:9007–9015. 10.1128/JVI.78.17.9007-9015.2004. 15308697 PMC506966

[30] Stertz S, Reichelt M, Spiegel M, Kuri T, Martinez-Sobrido L, Garcia-Sastre A, Weber F, Kochs G. 2007. The intracellular sites of early replication and budding of SARS-coronavirus. Virology 361:304–315. 10.1016/j.virol.2006.11.027. 17210170 PMC7103305

[31] Korber B, Fischer WM, Gnanakaran S, Yoon H, Theiler J, Abfalterer W, Hengartner N, Giorgi EE, Bhattacharya T, Foley B, Hastie KM, Parker MD, Partridge DG, Evans CM, Freeman TM, de Silva TI, Angyal A, Brown RL, Carrilero L, Green LR, Groves DC, Johnson KJ, Keeley AJ, Lindsey BB, Parsons PJ, Raza M, Rowland-Jones S, Smith N, Tucker RM, Wang D, Wyles MD, McDanal C, Perez LG, Tang H, Moon-Walker A, Whelan SP, LaBranche CC, Saphire EO, Montefiori DC. 2020. Tracking changes in SARS-CoV-2 spike: evidence that D614G increases infectivity of the COVID-19 virus. Cell 182:812.e19–827.e19. 10.1016/j.cell.2020.06.043. 32697968 PMC7332439

[32] Li Q, Wu J, Nie J, Zhang L, Hao H, Liu S, Zhao C, Zhang Q, Liu H, Nie L, Qin H, Wang M, Lu Q, Li X, Sun Q, Liu J, Zhang L, Li X, Huang W, Wang Y. 2020. The impact of mutations in SARS-CoV-2 spike on viral infectivity and antigenicity. Cell 182:1284.e9–1294.e9. 10.1016/j.cell.2020.07.012. 32730807 PMC7366990

[33] Cotter K, Stransky L, McGuire C, Forgac M. 2015. Recent insights into the structure, regulation, and function of the V-ATPases. Trends Biochem Sci 40:611–622. 10.1016/j.tibs.2015.08.005. 26410601 PMC4589219

[34] Bertrand P, Cote M, Zheng YM, Albritton LM, Liu SL. 2008. Jaagsiekte sheep retrovirus utilizes a pH-dependent endocytosis pathway for entry. J Virol 82:2555–2559. 10.1128/JVI.01853-07. 18094164 PMC2258929

[35] Mingo RM, Simmons JA, Shoemaker CJ, Nelson EA, Schornberg KL, D'Souza RS, Casanova JE, White JM. 2015. Ebola virus and severe acute respiratory syndrome coronavirus display late cell entry kinetics: evidence that transport to NPC1^+^ endolysosomes is a rate-defining step. J Virol 89:2931–2943. 10.1128/JVI.03398-14. 25552710 PMC4325712

[36] Kandeel M, Ibrahim A, Fayez M, Al-Nazawi M. 2020. From SARS and MERS CoVs to SARS-CoV-2: moving toward more biased codon usage in viral structural and nonstructural genes. J Med Virol 92:660–666. 10.1002/jmv.25754. 32159237 PMC7228358

[37] Shang J, Wan Y, Luo C, Ye G, Geng Q, Auerbach A, Li F. 2020. Cell entry mechanisms of SARS-CoV-2. Proc Natl Acad Sci U S A 117:11727–11734. 10.1073/pnas.2003138117. 32376634 PMC7260975

[38] McBride CE, Li J, Machamer CE. 2007. The cytoplasmic tail of the severe acute respiratory syndrome coronavirus spike protein contains a novel endoplasmic reticulum retrieval signal that binds COPI and promotes interaction with membrane protein. J Virol 81:2418–2428. 10.1128/JVI.02146-06. 17166901 PMC1865919

[39] Chen J, Kovacs JM, Peng H, Rits-Volloch S, Lu J, Park D, Zablowsky E, Seaman MS, Chen B. 2015. HIV-1 ENVELOPE. Effect of the cytoplasmic domain on antigenic characteristics of HIV-1 envelope glycoprotein. Science 349:191–195. 10.1126/science.aaa9804. 26113642 PMC4701381

[40] Chandrashekar A, Liu J, Martinot AJ, McMahan K, Mercado NB, Peter L, Tostanoski LH, Yu J, Maliga Z, Nekorchuk M, Busman-Sahay K, Terry M, Wrijil LM, Ducat S, Martinez DR, Atyeo C, Fischinger S, Burke JS, Slein MD, Pessaint L, Van Ry A, Greenhouse J, Taylor T, Blade K, Cook A, Finneyfrock B, Brown R, Teow E, Velasco J, Zahn R, Wegmann F, Abbink P, Bondzie EA, Dagotto G, Gebre MS, He X, Jacob-Dolan C, Kordana N, Li Z, Lifton MA, Mahrokhian SH, Maxfield LF, Nityanandam R, Nkolola JP, Schmidt AG, Miller AD, Baric RS, Alter G, Sorger PK, Estes JD, Andersen H, Lewis MG, Barouch DH. 2020. SARS-CoV-2 infection protects against rechallenge in rhesus macaques. Science 369:812–817. 10.1126/science.abc4776. 32434946 PMC7243369

[41] Yang ZY, Kong WP, Huang Y, Roberts A, Murphy BR, Subbarao K, Nabel GJ. 2004. A DNA vaccine induces SARS coronavirus neutralization and protective immunity in mice. Nature 428:561–564. 10.1038/nature02463. 15024391 PMC7095382

[42] Yu J, Li M, Wilkins J, Ding S, Swartz TH, Esposito AM, Zheng YM, Freed EO, Liang C, Chen BK, Liu SL. 2015. IFITM proteins restrict HIV-1 infection by antagonizing the envelope glycoprotein. Cell Rep 13:145–156. 10.1016/j.celrep.2015.08.055. 26387945 PMC4602366

